# Gene Expression in Gut Symbiotic Organ of Stinkbug Affected by Extracellular Bacterial Symbiont

**DOI:** 10.1371/journal.pone.0064557

**Published:** 2013-05-14

**Authors:** Ryo Futahashi, Kohjiro Tanaka, Masahiko Tanahashi, Naruo Nikoh, Yoshitomo Kikuchi, Bok Luel Lee, Takema Fukatsu

**Affiliations:** 1 Bioproduction Research Institute, National Institute of Advanced Industrial Science and Technology (AIST), Tsukuba, Ibaraki, Japan; 2 Department of Liberal Arts, The Open University of Japan, Chiba, Chiba, Japan; 3 Bioproduction Research Institute, Hokkaido Center, National Institute of Advanced Industrial Science and Technology (AIST), Sapporo, Hokkaido, Japan; 4 College of Pharmacy, Pusan National University, Geumjeong-gu, Busan, Korea; University Of Montana – Missoula, United States of America

## Abstract

The bean bug *Riptortus pedestris* possesses a specialized symbiotic organ in a posterior region of the midgut, where numerous crypts harbor extracellular betaproteobacterial symbionts of the genus *Burkholderia*. Second instar nymphs orally acquire the symbiont from the environment, and the symbiont infection benefits the host by facilitating growth and by occasionally conferring insecticide resistance. Here we performed comparative transcriptomic analyses of insect genes expressed in symbiotic and non-symbiotic regions of the midgut dissected from *Burkholderia*-infected and uninfected *R. pedestris*. Expression sequence tag analysis of cDNA libraries and quantitative reverse transcription PCR identified a number of insect genes expressed in symbiosis- or aposymbiosis-associated patterns. For example, genes up-regulated in symbiotic relative to aposymbiotic individuals, including many cysteine-rich secreted protein genes and many cathepsin protease genes, are likely to play a role in regulating the symbiosis. Conversely, genes up-regulated in aposymbiotic relative to symbiotic individuals, including a chicken-type lysozyme gene and a defensin-like protein gene, are possibly involved in regulation of non-symbiotic bacterial infections. Our study presents the first transcriptomic data on gut symbiotic organ of a stinkbug, which provides initial clues to understanding of molecular mechanisms underlying the insect-bacterium gut symbiosis and sheds light on several intriguing commonalities between endocellular and extracellular symbiotic associations.

## Introduction

The majority of insects are associated with microbial symbionts within their alimentary tract, body cavity and/or cells, and they are often benefited from the symbiosis for their growth, viability and fecundity. Hence, understanding of the mechanisms of establishment, maintenance and fitness consequences of such host-symbiont associations is of fundamental importance [1]–[6].

Transcriptomic analyses of the bacteriomes, which are specialized insect organs consisting of bacteriocytes for harboring microbial symbionts, have been conducted for aphid-*Buchnera*, weevil-*Sodalis* and bedbug-*Wolbachia* endosymbiotic associations of obligate nature [7]–[12]. Comparative transcriptomics of symbiont-infected and uninfected individuals have been applied to diverse arthropods and their facultative endosymbionts like *Wolbachia*, *Cardinium* and *Serratia* [13]–[18]. These studies show that the expression of immune-related genes such as lysozyme genes and antimicrobial peptide genes is often affected by endosymbiont infection in a tissue-specific manner. Notably, a number of cysteine-rich secreted proteins are highly expressed in the aphid bacteriocytes [7], which has been also known in plant symbioses such as legume-*Rhizobium* associations [19].

Within the insect suborder Heteroptera, more than 12,500 species of true bugs or stinkbugs constitute the infraorder Pentatomomorpha [20]. Besides relatively few predacious and mycophagous species, most of the phytophagous stinkbugs possess a specialized symbiotic region in the posterior midgut. The gut symbiotic organ is equipped with a number of sac- or tube-like crypts, whose lumen harbors specific extracellular symbiotic bacteria. In general, these gut symbionts significantly benefit their host stinkbugs: symbiont-deprived insects suffer retarded growth, increased nymphal mortality and/or adult sterility [3], [21], [22]. To our knowledge, no transcriptomic studies have been conducted on such insect symbiotic organs associated with specific extracellular symbionts.

The bean bug *Riptortus pedestris* (Hemiptera: Heteroptera: Alydidae) possesses the midgut symbiotic organ with numerous crypts, whose lumen is full of betaproteobacterial extracellular symbionts of the genus *Burkholderia* [23]. The gut symbiont is not essential but beneficial for the host stinkbug: uninfected insects are able to become adult and reproduce, but their growth rate and body size are significantly reduced in comparison with symbiotic insects [24]. In addition to the putative nutritional role, some *Burkholderia* strains are capable of degrading organophophorus insecticides, thereby making their host insects resistant to the toxic chemicals [25]. The *Riptortus*-*Burkholderia* gut symbiosis is regarded as a promising model system for insect symbiosis studies in that (i) the symbiont is easily culturable on standard microbiological media, which is exceptional among insect symbiotic bacteria of beneficial nature, (ii) the symbiont is orally acquired by young nymphal stinkbugs from the soil environment every generation, (iii) both symbiotic and aposymbiotic insects are able to become adult and reproduce, and (iv) RNA interference of the host gene expression is feasible [24], [26], [27]. Owing to these features, symbiotic and aposymbiotic insects are easily compared experimentally.

In this study, we constructed expression sequence tag (EST) libraries of symbiotic and non-symbiotic midgut regions dissected from symbiotic and aposymbiotic individuals of *R. pedestris*, which provide the first transcriptomic data on gut symbiotic organ of a stinkbug, and unveil a number of insect genes including lysozyme gene, defensin-like protein gene, cathepsin protease genes, and cysteine rich secreted protein genes that are potentially involved in symbiotic interactions between the *Burkholderia* symbiont and the *Riptortus* host.

## Results

### Gut morphology of symbiotic and aposymbiotic insects

**Figure 1:**
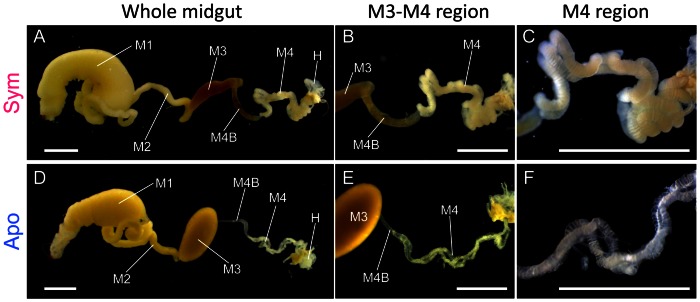
Dissected midgut of *R. pedestris* three days after fifth instar molt. (A–C) Midgut of symbiotic insect. (D–E) Midgut of aposymbiotic insect. Abbreviations: M1, midgut first region; M2, midgut second region; M3, midgut third region; M4, midgut fourth region with crypts; M4B, anterior bulb of midgut fourth section; H, hindgut. Bars show 2 mm.

Figure 1 shows the midgut of *R. pedestris* consisting of several morphologically distinct regions: from anterior to posterior, stomach-like midgut first region (M1); tubular midgut second region (M2); expanded sac-like midgut third region (M3); and midgut fourth region (M4) with numerous crypts whose lumen is full of symbiotic *Burkholderia* cells. Between M3 and M4, there is a slightly enlarged, tubular portion, called anterior bulb of M4 or M4 bulb (M4B), which bears no crypts but contains the symbiotic bacteria [25], [28]. Among the midgut regions, M3, M4 and M4B exhibited remarkable morphological differences between symbiotic insects and aposymbiotic insects. M4 and M4B were enlarged in symbiotic insects (Fig. 1A–C), whereas the midgut regions were atrophied in aposymbiotic insects (Fig. 1D–F). By contrast, M3 was larger in aposymbiotic insects (Fig. 1D and E) than in symbiotic insects (Fig. 1A and B). These morphological differences were consistent across all individuals we examined.

### Construction of midgut EST datasets

For a symbiotic insect and an aposymbiotic insect of the same isofemale line, we constructed cDNA libraries for each of the midgut region M3, M4B or M4. We used individuals at three days after fifth instar molt in this study because morphological differences of the midgut between symbiotic insects and aposymbiotic insects were conspicuous and suitable for dissecting each midgut region. In total, 6,924 clones were sequenced for the cDNA libraries of the symbiotic and aposymbiotic insects (DDBJ accession numbers HX275191-HX282114) (Table 1). From these ESTs, sequences corresponding to insect ribosomal RNA (DDBJ accession number AB725684), insect mitochondrial DNA (DDBJ accession number EU427344), and symbiont genes based on the draft genome sequence of the *Burkholderia* symbiont (Nikoh N et al., unpublished data) were eliminated at the criterion of E-value <10^−20^ under the BLASTn algorithm, which yielded 6,234 ESTs. These ESTs were subjected to automatic clustering by the Phred/Phrap/Consed software package (http://bozeman.mbt.washington.edu), and subsequently each cluster was inspected and corrected manually by dividing and reassembling putative chimeric sequences, which yielded 1,863 non-redundant EST clusters (Tables S1 and S2). Of these, 41 clusters were regarded either as isoforms or as premature forms of other clusters judging from their sequence identities (Table S2). Excluding these clusters, we obtained 1,822 non-redundant EST clusters/singletons (DDBJ accession numbers AB591382, AK416867- AK418687) (Table 1). Each of the clusters was assigned a serial identification number in the order of number of clones appearing in the total EST dataset (Table S1). Of the 1,822 clusters, 1,624 contained ORFs encoding predicted proteins no shorter than 50 amino acids, of which 1,194 and 1,173 exhibited significant sequence similarities to protein sequences of the fruit fly *Drosophila melanogaster* (Flybase ver. 5.42) and the aphid *Acyrthosiphon pisum* [29], respectively, at the cutoff threshold E-value of *P*<1e^−10^ by BLASTP search (Table S1).

**Table 1:** Summary of EST data sets.

	SymM3	SymM4B	SymM4	ApoM3	ApoM4B	ApoM4	Total
Number of clones	1,033	1,285	1,458	917	1,111	1,120	6,924
Mitochondrial DNA	67	49	38	48	43	31	276
Ribosomal RNA	195	46	26	65	42	31	405
*Burkholderia* genes	0	1	8	0	0	0	9
Analyzed ESTs	771	1,189	1,386	804	1,026	1,058	6,234
No. of unique clusters	440	367	532	482	505	487	1,822

### Gene ontology terms of EST datasets from symbiotic and aposymbiotic insects

**Figure 2:**
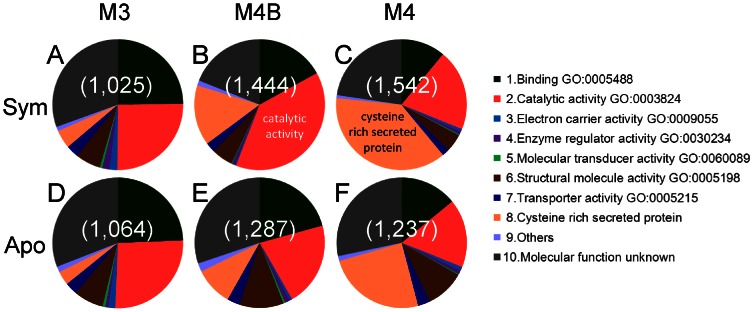
Gene Ontology (GO) molecular function terms assigned to the midgut EST data of *R. pedestris*. Ratio of number of EST clones representing each GO term per total number of EST clones is shown as each pie graph division. (A–C) Symbiotic insect. (D–F) Aposymbiotic insect. Number of total EST clones is indicated in parentheses. Because one cluster can be associated with more than one GO term, total number of EST clones shown in parentheses may be different from those shown in Table 1. Abbreviations: Sym, symbiotic insect; Apo, aposymbiotic insect; M3, midgut third region; M4: midgut fourth region with crypts; M4B, anterior bulb of midgut fourth section.

For each of the midgut EST datasets obtained from the M3, M4B and M4 regions of the symbiotic and aposymbiotic insects, the gene clusters were categorized into gene ontology (GO) molecular function terms that had been applied to *Drosophila* proteins based on Flybase ver. 5.42 (Fig. 2, Table S3). Besides the conventional GO terms, we adopted an additional category under the following criteria. Using the SignalP 4.0 program, we identified 465 genes with putative signal peptides, whose sequences are shown in Table S1. Among them, 97 genes had six or more cysteine residues and exhibited no sequence similarity to *Drosophila* and *Acyrthosiphon* genes. We categorized these genes as “cysteine-rich secreted protein” in this study (Fig. 2, Table S3).

For the M3 region, the composition of GO terms of the symbiotic insect was quite similar to that of the aposymbiotic insect (Fig. 2A and D), which probably reflects the fact that the M3 region is not infected with the symbiont. For the M4B and M4 regions where the symbiont is localized, by contrast, the compositions of GO terms were remarkably different between the symbiotic insect and the aposymbiotic insect. In particular, the category “catalytic activity” was identified more frequently in the M4B region of the symbiotic insect than in the M4B region of the aposymbiotic insect (Fig. 2B and E), and the category “cysteine-rich secreted protein” was more represented in the M4 region of the symbiotic insect than in the M4 region of the aposymbiotic insect (Fig. 2C and F).

### Dominant ESTs in the midgut cDNA libraries

In the midgut cDNA libraries, 6 genes were highly represented by more than 100 EST clones, and 20 genes were frequently represented by more than 30 EST clones (Table S1). Among them, 9 genes encoded cysteine-rich secreted proteins, 7 genes represented non-cysteine-rich, unknown secreted proteins, 3 genes represented cathepsin L proteases, 3 genes represented ferritin subunit proteins, and the remaining 4 genes encoded cathepsin B, zinc carboxypeptidase proteases, actin and c-type lysozyme, respectively (Table S1).

### Symbiosis- and aposymbiosis-specific ESTs in the midgut regions

In the EST datasets, we identified 11 candidate symbiosis-specific genes that were represented by no less than 10 EST clones and detected exclusively in the symbiotic insect (Table 2). These genes exhibited the following patterns: (i) ten genes were preferentially expressed in the M4 and/or M4B regions of the symbiotic insect (except for glyoxal oxidase [Rped-0100]), (ii) six genes preferentially expressed in the M4 region encoded cysteine-rich secreted proteins (except for cathepsin L [Rped-0047] and unknown secreted protein [Rped-0090]), and (iii) two genes preferentially expressed in the M4B region encoded enzymes such as zinc carboxypeptidase [Rped-0023] and gpi-anchor transamidase [Rped-0031] (Table 2). These expression patterns were the main reason for different GO terms composition between symbiotic and aposymbiotic insects (Fig. 2).

**Table 2:** Symbiosis-associated genes identified in the EST analysis.

Cluster ID	No. of clones	Annotation	SymM3	SymM4B	SymM4	ApoM3	ApoM4B	ApoM4	qRT-PCR* ^1^
Rped-0008	62	Cysteine-rich secreted protein	1	0	61	0	0	0	*P*<0.05 (M4)
Rped-0023	33	Zinc carboxypeptidase	0	32	1	0	0	0	*P*<0.001 (M4)
Rped-0031	24	gpi-anchor transamidase	0	21	3	0	0	0	N.S.
Rped-0043	19	Cysteine-rich secreted protein	0	2	17	0	0	0	*P*<0.05 (M4)
Rped-0047	18	CathepsinL	0	0	18	0	0	0	*P*<0.001 (M4)
Rped-0061	15	Cysteine-rich secreted protein	0	0	15	0	0	0	N.S.
Rped-0077	12	Cysteine-rich secreted protein	0	0	12	0	0	0	N.S.
Rped-0090	11	Unknown secreted protein	0	0	11	0	0	0	*P*<0.001 (M4)
Rped-0095	10	Cysteine-rich secreted protein	0	0	10	0	0	0	N.S.
Rped-0100	10	Glyoxal oxidase	10	0	0	0	0	0	*P*<0.05 (M1,M2,M3)
Rped-0106	10	Cysteine-rich secreted protein	0	0	10	0	0	0	*P*<0.05 (M4)

**Figure 3:**
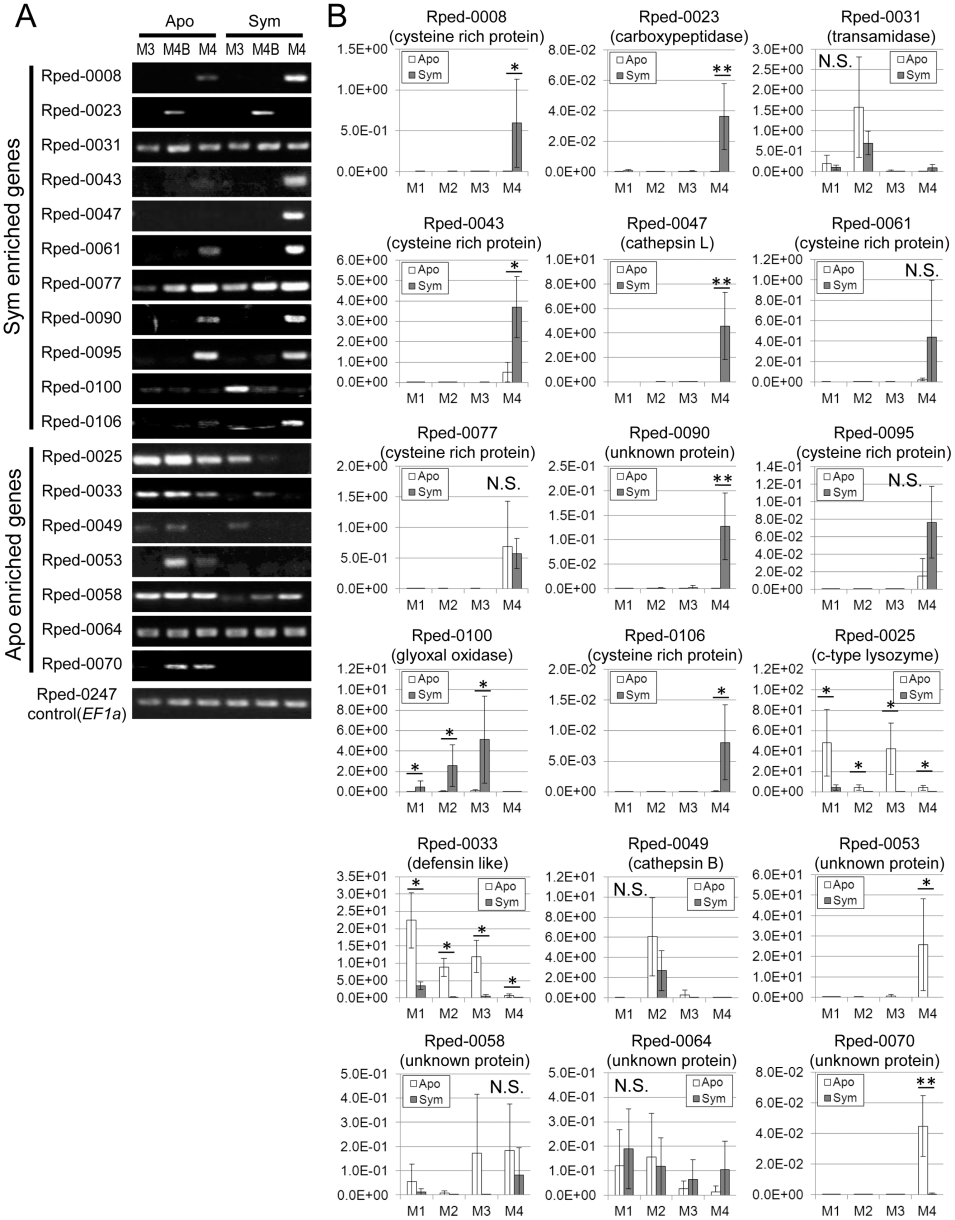
Relative expression levels of symbiosis- and aposymbiosis-associated gene candidates in *R. pedestris*. (A) Semi-quantitative RT-PCR of candidate genes. Abbreviations are the same as in Figure 2. Elongation factor 1 alpha (*EF1a*) gene (Rped-0247) was used as an internal control. (B) Relative expression levels of candidate genes in four midgut parts, namely M1, M2, M3, and M4 (plus M4B). The expression levels were evaluated by quantitative RT-PCR in terms of each gene cDNA copies per *EF1α* cDNA copy. Means and standard deviations (n = 8) are shown. Statistically significant differences between aposymbiotic and symbiotic insects in each midgut region are shown by asterisks (t test; *, *P*<0.05; **, P<0.001). N.S. indicates no significant difference.

* 1: Statistically significant differences between aposymbiotic and symbiotic insects in each midgut region by quantitative RT-PCR (*t* test; n = 8, see Figure 3B). N.S., not significant.

We also identified 7 candidate aposymbiosis-specific genes that were represented by no less than 10 EST clones and detected exclusively in the aposymbiotic insect (Table 3). These genes exhibited the patterns that (i) six genes were preferentially expressed in the M4B region (except for cathepsin B protease [Rped-0049]), (ii) two genes encoded defense-related proteins such as c-type lysozyme [Rped-0025] and defensin-like protein [Rped-0033], (iii) these defense-related proteins were expressed not only in the M4B region but also in the M3 region, and (iv) the other genes encoded unknown proteins (Table 3).

**Table 3:** Aposymbiosis-associated genes identified in the EST analysis.

Cluster ID	No. of clones	Annotation	SymM3	SymM4B	SymM4	ApoM3	ApoM4B	ApoM4	qRT-PCR* ^1^
Rped-0025	32	c-type lysozyme	0	0	0	5	26	1	P<0.05 (M1,M2,M3,M4)
Rped-0033	23	Defensin-like protein	0	0	0	11	9	3	*P*<0.05 (M1,M2,M3,M4)
Rped-0049	18	Cathepsin B	0	0	0	18	0	0	N.S.
Rped-0053	17	Unknown secreted protein	0	0	0	0	17	0	*P*<0.05 (M4)
Rped-0058	16	Unknown protein	0	0	0	0	14	2	N.S.
Rped-0064	15	Unknown protein	0	0	0	6	7	2	N.S.
Rped-0070	14	Unknown secreted protein	0	0	0	0	8	6	*P*<0.001 (M4)

* 1: Statistically significant differences between aposymbiotic and symbiotic insects in each midgut region by quantitative RT-PCR (*t* test; n = 8, see Figure 3B). N.S., not significant.

### Expression analysis of symbiosis- and aposymbiosis-associated genes

Genes representing 11 symbiosis-specific ESTs and 7 aposymbiosis-specific ESTs were subjected to semi-quantitative RT-PCR of M3, M4B, and M4 regions (Fig. 3A) and real-time quantitative RT-PCR of M1, M2, M3, and M4 (plus M4B) regions of symbiotic and aposymbiotic insects (Fig. 3B).

Of the 11 candidate symbiosis-associated genes, six genes, namely Rped-0008 (cysteine-rich secreted protein), Rped-0023 (carboxypeptidase), Rped-0043 (cysteine-rich secreted protein), Rped-0047 (cathepsin L protease), Rped-0090 (unknown secreted protein) and Rped-0106 (cysteine-rich secreted protein), exhibited specifically and significantly increased expression in the M4 plus M4B region of symbiotic insects relative to the same region of aposymbiotic individuals, whereas one gene, Rped-0100 (glyoxal oxidase), exhibited significantly higher expression levels in the M1, M2 and M3 regions of symbiotic insects but little expression in the M4 plus M4B region. Rped-0061, Rped-0077 and Rped-0095 (all cysteine-rich secreted proteins) were preferentially expressed in the M4 plus M4B region, but the differences between symbiotic insects and aposymbiotic insects were not statistically significant (Fig. 3B).

Of the 7 candidate aposymbiosis-associated genes, two genes, namely Rped-0025 (c-type lysozyme) and Rped-0033 (defensin-like protein), exhibited consistently and significantly higher expression levels in all the midgut regions of aposymbiotic insects, whereas two genes, Rped-0053 and Rped-0070 (both unknown proteins), were specifically and highly expressed in the M4 plus M4B region of aposymbiotic insects (Fig. 3B).

### Aposymbiosis-associated and other lysozyme genes

Diverse lysozyme genes are phylogenetically classified into chicken (c-), goose (g-), invertebrate (i-), bacterial and other types [30]. The lysozyme gene Rped-0025, which was highly expressed in the midgut of aposymbiotic insects but scarcely expressed in the midgut of symbiotic insects (Fig. 3; Table 3), was placed in the c-type lysozyme clade (Fig. S1).

Besides the highly-expressed and aposymbiosis-associated c-type lysozyme gene Rped-0025 (32 ESTs), two lysozyme transcripts, representing a bacterial type lysozyme gene Rped-0028 (28 ESTs) and a c-type lysozyme gene Rped-0069 (14 ESTs), were identified (Fig. S1), although expression of these genes were found in both the symbiotic and aposymbiotic insects (Table S4).

### Aposymbiosis-associated defensin-like gene

**Figure 4:**
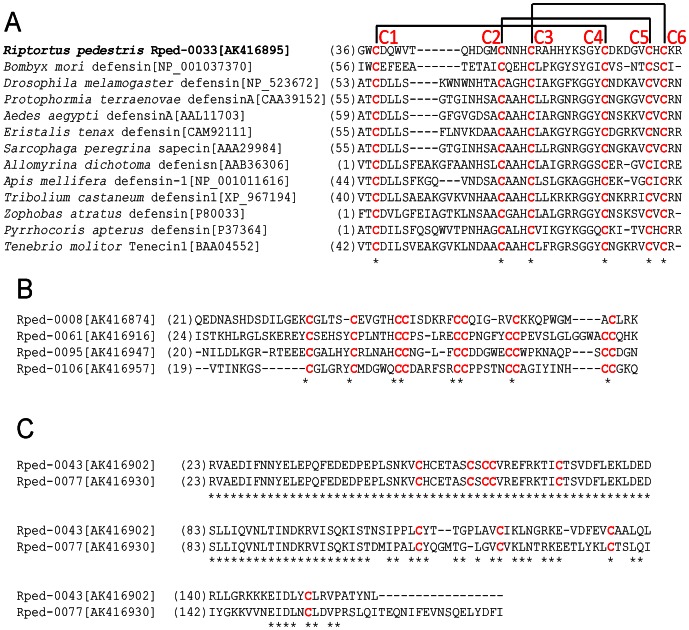
Cysteine-rich protein genes identified in the EST data of *R. pedestris*. (A) Amino acid sequence of defensin-like gene of *R. pedestris* compared with sequences of defensins from other insects. (B) Amino acid sequences of four cysteine-rich secretion proteins of short type. (C) Amino acid sequences of two cysteine-rich secretion proteins of long type. Conserved cysteine residues are highlighted in red. Estimated disulphide bridges are shown by solid line in (A). Accession numbers are in brackets, and first amino acid positions are in parentheses.

Insect defensins are cationic antimicrobial peptides consisting of 34–46 amino acid residues with molecular masses ranging from 2 to 6 kDa, in which the positions of six cysteine residues forming three intramolecular disulfide bridges are conserved and essential for expressing antimicrobial activities [31], [32]. The defensin-like gene Rped-0033, which was highly expressed in the midgut of aposymbiotic insects but scarcely expressed in the midgut of symbiotic insects (Fig. 3; Table 3), retained six cysteine residues but the other regions were not similar to conventional defensins (Fig. 4A).

Besides the highly-expressed and aposymbiosis-associated defensin-like gene Rped-0033 (23 ESTs), no other defensin-like gene was detected in the cDNA libraries, although multiple defensin-like genes have been identified in other heteropteran bugs [33], [34].

### Symbiosis-associated and other cysteine-rich secreted protein genes

Recent accumulation of genomic and transcriptomic data revealed that cysteine-rich secreted proteins, which are structurally similar to defensins in that they are cationic secreted peptides with 6–8 conserved cysteine residues that are predicted to form intramolecular disulfide bridges, are ubiquitously found across diverse organisms [35]. In the cDNA libraries of *R. pedestris*, we identified several cysteine-rich secreted protein genes whose expression patterns were strongly associated with the midgut region M4 of symbiotic insects (Fig. 3; Table 2). Some of them encoded relatively small peptides with around 70–90 residues (Fig. 4B), while others encoded larger peptides with about 150–170 residues (Fig. 4C).

In total, as many as 97 cysteine-rich secreted protein genes (including the symbiosis-associated genes listed in Table 2) were identified in the cDNA libraries, which accounted for 5.3% (97/1,822) of the genes and 21.0% (1,307/6,234) of the ESTs identified in the cDNA libraries. Notably, even when their expression was not associated with symbiotic status, many, if not all, of them exhibited preferential expression in the midgut M4 and/or M4B regions (ex. Rped-0001, Rped-0003, Rped-0009, Rped-0017, Rped-0026, Rped-0035, Rped-0037, Rped-0039, Rped-0048, Rped-0056, and others) (Table S5). These genes exhibited no significant sequence similarities to cysteine-rich secreted proteins of other organisms deposited in the public DNA and protein databases.

### Symbiosis-associated and other cathepsin protease genes

**Figure 5:**
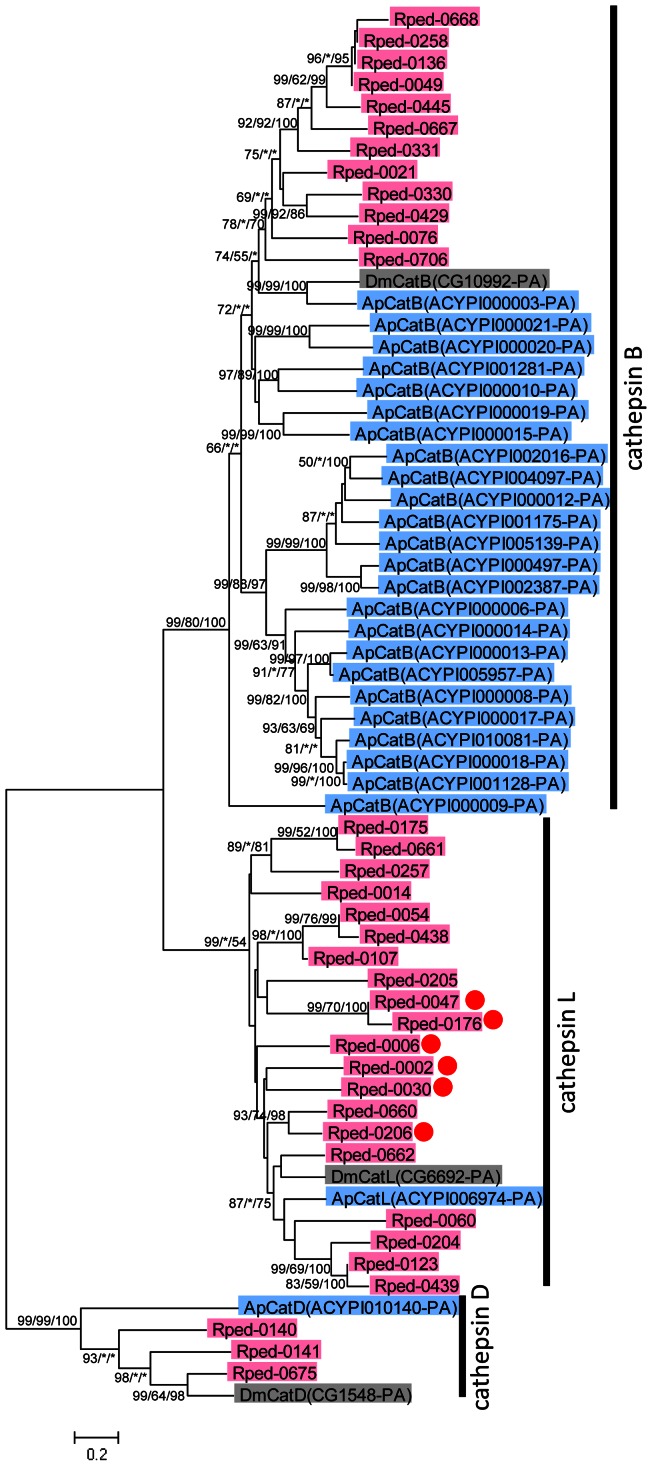
Diversity of cathepsin protease genes identified in the EST data of *R. pedestris*. A neighbor-joining phylogeny inferred from 428 aligned amino acid sites is shown, while maximum likelihood and Bayesian phylogenies exhibited substantially the same topologies. On each node, statistical support values are indicated in the order of [bootstrap value of neighbor-joining]/[bootstrap value of maximum likelihood]/[posterior probability of Bayesian]. Asterisks indicate support values lower than 50%. Clades of cathesin B, cathepsin L and cathepsin D are indicated on the right side. Genes of *R. pedestris*, *A. pisum* and *D. melanogaster* are colored in red, blue and gray, respectively. Red circles indicate genes preferentially expressed in symbiotic insects (EST clones more than tenfold in symbiotic insect and no less than 5 EST clones).

Several protease genes, namely Rped-0023 encoding zinc carboxypeptidase and Rped-0047 encoding cathepsin L protease, were highly and specifically expressed in the midgut region M4 of symbiotic insects (Fig. 3; Table 2). In the cDNA libraries of *R. pedestris*, notably, we identified a large number of cathepsin protease genes: 21 cathepsin L genes, 13 cathepsin B genes, and 3 cathepsin D genes (Table S6). In the genome of the fruit fly *D. melanogaster*, cathepsin L, cathepsin B and cathepsin D genes are all single-copied [36]. On the other hand, in the genome of the aphid *A. pisum*, cathepsin B genes are amplified to 27 copies via repeated gene duplications, whereas cathepsin L and cathepsin D genes are single-copied [29], [37]. Molecular phylogenetic analysis showed that (i) cathepsin B genes, cathepsin L genes and cathepsin D genes of *R. pedestris* constitute distinct monophyletic groups in the cathepsin phylogeny, respectively, (ii) cathepsin B genes of *R. pedestris* formed a cluster distinct from the cluster of cathepsin B genes of *A. pisum*, (iii) thus, cathepsin genes were probably amplified in the stinkbug lineage and in the aphid lineage independently, and (iv) cathepsin L genes were also amplified in the stinkbug lineage (Fig. 5).

### Symbiosis-associated expression and activity of cathepsin L proteases

**Figure 6:**
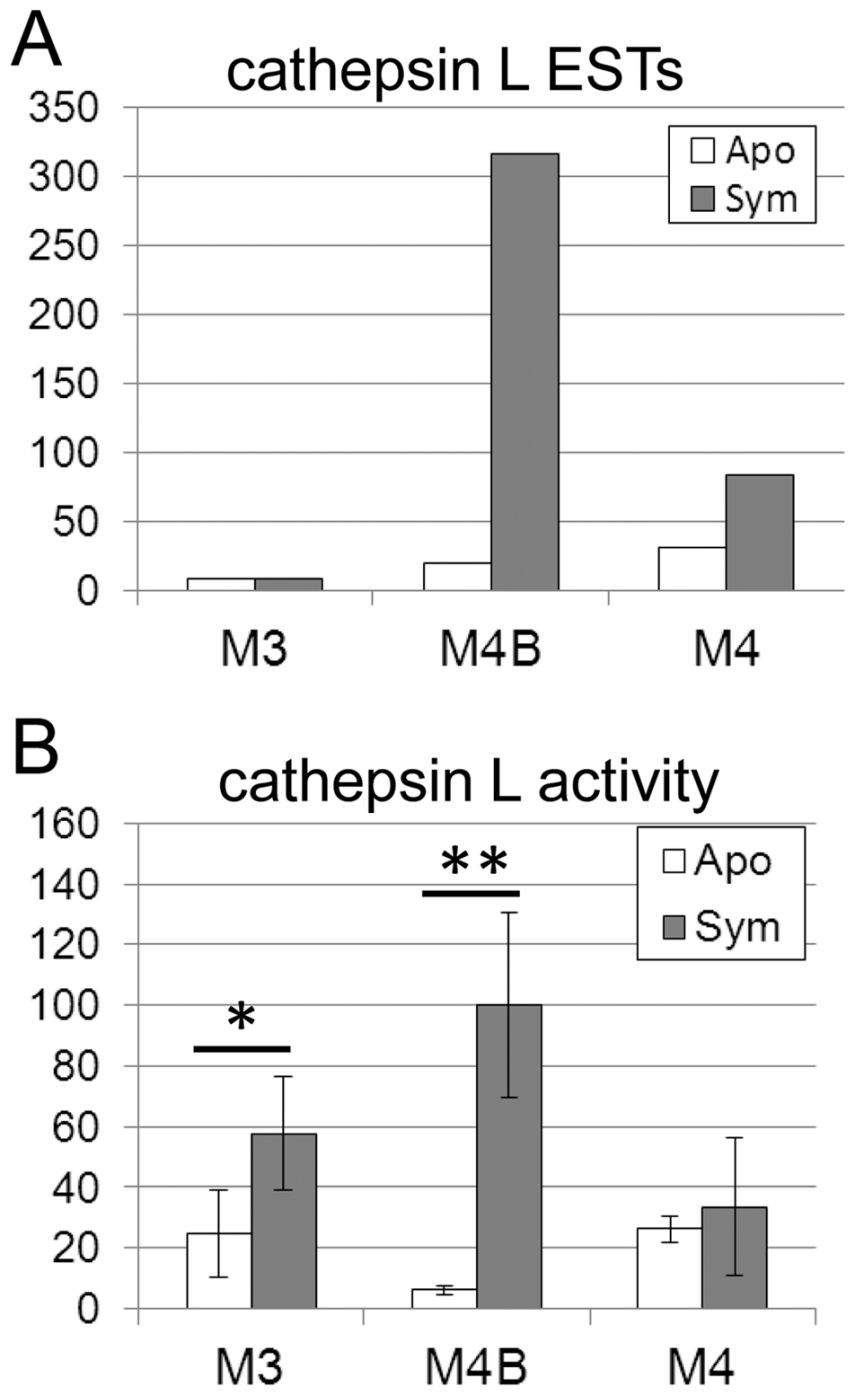
Expression and activity of cathepsin L proteases in midgut regions of *R. pedestris*. (A) Total number of EST clones representing cathepsin L genes in the midgut EST libraries. (B) Relative enzymatic activities of cathepsin L proteases in extracts of the midgut regions. Means and standard deviations (n = 10) are shown. Statistically significant differences are indicated by asterisks (t test; *, *P*<0.05; **, *P*<0.001). Abbreviations are as in Figure 2.

In the cDNA libraries of *R. pedestris*, cathepsin L genes accounted for as much as 7.5% of the total ESTs (466/6,234), whereas cathepsin B genes and cathepsin D genes represented only 1.4% (90/6,234) and 0.2% (15/6,234), respectively. In particular, the cathepsin L genes Rped-0002 (194 ESTs), Rped-0006 (101 ESTs) and Rped-0030 (24 ESTs) were notable in that they are not only highly represented in the cDNA libraries (319/6,234 = 5.1%) but also exclusively expressed in the midgut M4B region of symbiotic insects (Table S6). Consequently, most of the cathepsin L ESTs were represented in the cDNA library of the M4B region of the symbiotic insect (Fig. 6A).

By making use of the synthetic fluorescent substrate, Z-Val-Val-Arg-MCA, that is specifically hydrolyzed by cathepsin L-like proteases [38], we enzymatically measured cathepsin L activities in homogenates of dissected M3, M4B and M4 regions of symbiotic and aposymbiotic insects. In agreement with the EST results (Fig. 6A), the highest cathepsin L activities were found in the M4B region of symbiotic insects, which were significantly higher than the activities in the M4B region of aposymbiotic insects (Fig. 6B). Meanwhile, being unexpected from the EST results (Fig. 6A), considerable cathepsin L activities were also detected in the M3 region of both symbiotic and aposymbiotic insects, and the activities in symbiotic insects were significantly higher than the activities in aposymbiotic insects (Fig. 6B).

### Symbiosis- and aposymbiosis-associated secreted protein genes of unknown function

Rped-0053 and Rped-0070 were specifically expressed in the M4 region of aposymbiotic insects (Table 3; Fig. 3), whereas Rped-0090 was specifically expressed in the M4 region of symbiotic insects (Table 2; Fig. 3). These genes were with signal peptide sequences at their 5′ end, and exhibited no sequence similarity to known proteins in the public DNA and protein databases. Instead of being cysteine-rich, Rped-0053, Rped-0070 and Rped-0090 were lysine-, serine- and leucine-rich proteins, respectively (Table S1).

## Discussion

In this study, we constructed EST libraries of symbiotic and non-symbiotic regions of the midgut dissected from symbiotic and aposymbiotic insects of *R. pedestris*, and the EST data revealed a number of intriguing candidate genes whose expression patterns are correlated to symbiosis/aposymbiosis with the *Burkholderia* gut symbiont. The symbiosis-related transcriptomic data provide valuable basic information as well as initial clues to the molecular mechanisms underlying the host-symbiont interactions. Hereafter, we discuss potential biological roles of the symbiosis- and aposymbiosis-associated genes of *R. pedestris* identified in this study. Needless to say, the arguments based on the EST data and previous relevant literatures are speculative, but they will provide working hypotheses directing toward future experimental studies.

### Identification of many cysteine-rich secretion protein genes: candidate effector molecules involved in host-symbiont interactions

Identification of 97 cysteine-rich secretion protein genes, many of which are preferentially expressed in the symbiotic midgut regions and some of which are expressed in a symbiosis-associated manner, comprises the most interesting finding in this study, on the ground that recent studies have highlighted biological importance of cysteine-rich secreted proteins in plant and insect endosymbiotic systems. In the legume-*Rhizobium* nitrogen-fixing symbiosis, many cysteine-rich secreted protein genes are preferentially expressed in root nodules, and at least some of them exhibit antimicrobial activities *in vitro* and induce irreversible differentiation of the symbiont cells into bacteroids *in planta* [19], [39]. In the aphid-*Buchnera* nutritional symbiosis, a number of cysteine-rich secreted protein genes are expressed in a bacteriocyte-specific manner, although their biological roles are elusive [7]. Furthermore, recent accumulation of genomic and transcriptomic data has revealed abundant occurrences of cysteine-rich secreted proteins in other organisms including *Arabidopsis thaliana* [40] and other plants [41], and also corals [42]. These relatively short peptides are structurally related to antimicrobial peptides like defensins in that they are cysteine-rich and cationic, and many of them are thought to have antimicrobial activities [43]. Identification of many cysteine-rich secretion protein genes expressed in the gut symbiotic organ of *R. pedestris* highlights an unexpected molecular commonality among endocellular and extracellular symbiotic associations in plants and insects.

Biological functions of the cysteine-rich proteins in the *Riptortus*-*Burkholderia* gut symbiosis are currently unknown. In the legume-*Rhizobium* symbiosis, the nodule-specific cysteine-rich proteins target the bacterial membrane and cytosol within the symbiosome, and act as plant effectors to direct the bacteroids into a terminally differentiated state [19], [44]. It is conceivable, although speculative, that similarly, the cysteine-rich proteins may be secreted from the intestinal epithelial cells into the gut lumen, and act on proliferation and/or physiology of the symbiont cells. Experimental studies *in vivo* (suppression of the cysteine-rich proteins by RNA interference) and *in vitro* (incubation of the symbiont cells with the cysteine-rich proteins) are to be conducted to verify this hypothesis.

### Aposymbiosis-associated expression of lysozyme and defensin-like genes: possible biological role in the context of symbiosis

Lysozymes are the enzymes that destroy bacterial cell walls by degrading peptideglycans, thereby showing antibacterial activities and playing important roles in defense against bacterial infections [45]. Conventionally, lysozymes have been, together with an array of antimicrobial peptides, regarded as inducible bactericidal proteins that are highly expressed in response to microbial infections and accumulate in the insect hemolymph [31], [46]. However, the lysozyme gene Rped-0025 and the defensin-like gene Rped-0033 of *R. pedestris* were highly expressed in aposymbiotic insects but scarcely expressed in symbiotic insects (Fig. 3; Table 3), suggesting a unique regulation of these defense-related genes in the context of host-symbiont interactions. It should be noted that, recently, a variety of correlations between lysozyme gene expression and symbiont infection have been reported in other symbiotic systems: expression of a c-type lysozyme gene is down-regulated in the ovary of *Wolbachia*-infected parasitic wasp *Asobara tabida* [16]; expression of an i-type lysozyme gene is down-regulated in *Wolbachia*-infected pill bug *Armadillidium vulgare* [15]; in the grain weevils *Sitophilus zeamais* and *S. oryzae*, expression of an i-type lysozyme gene is down-regulated in the bacteriocytes harboring *Sodalis*-allied symbiotic bacteria endocellularly [10], [11]; and in the pea aphid *Acyrthosiphon pisum*, strikingly, two i-type lysozyme genes are specifically expressed in the bacteriocytes harboring *Buchnera*, which represent the most abundant transcripts in the symbiotic cells [8]. Meanwhile, in the *Sitophilus* weevils, permanent infection of bacteriocytes with *Sodalis-*allied primary endosymbiont leads to up-regulation of an antimicrobial peptide, coleoptericin-A, whose function is to restrict the endosymbiont infection to the bacteriocytes [47]. Coleoptericin-A contains no cysteine residue, which is different from the defensin-like proteins [32].

Biological function of the aposymbiosis-associated lysozyme and defensin-like gene in the midgut of *R. pedestris* is currently elusive. In saprophagous insects like *D. melanogaster*, a part of amplified lysozyme genes are preferentially expressed in the midgut [48], [49], which are suggested to function for digesting bacteria-rich fermented foods [46]. Digestive roles of gut-associated lysozyme genes have also been suggested for the house fly *Musca domestica* [50], mosquitoes [51] and termites [52]. However, it seems unlikely that the midgut lysozyme of *R. pedestris* plays a digestive role because (i) the food of the stinkbug, plant sap, is not bacteria-rich, (ii) food digestion must be necessary for both symbiotic and aposymbiotic insects, and (iii) if the symbiotic bacteria in the midgut are digested and utilized, suppressed expression of the lysozyme gene in symbiotic insects does not make sense.

In holometabolous insects, up-regulated immune functions including lysozyme production have been detected in the midgut of mature larvae before pupation, which are presumably vulnerable to bacterial infections during the radical developmental reorganization of metamorphosis [49], [53]–[55]. In several hemimetabolous insects and ticks, up-regulated lysozyme expression is observed immediately after molting [56], [57]. In the midgut epithelial cells of the tobacco hornworm *Manduca sexta*, lysozyme granules are stored and released into the gut lumen just before metamorphosis [53]. Here, these gut lysozymes may have a defensive role in the course of insect development, particularly against potentially virulent gut microbes at the immune-compromised metamorphosis and molting stages [46].

Considering the similar expression patterns of lysozyme gene and defensin-like gene (Fig. 3B), we suggest the possibility that the lysozyme and defensin-like gene product may be involved in suppression of improper bacterial infections in the midgut. In this context, it may be relevant that synergistic bactericidal effects of lysozyme and other antimicrobial peptides including defensin have been reported [58], [59]. Whether expression of these and other defense-related genes are induced by infection with non-symbiotic bacteria in *R. pedestris* is of interest and deserves future studies.

### Over-expression of cathepsin L protease genes in the midgut M4B region: candidate molecules involved in regulation over symbiont population

Cathepsins are lysosomal acidic proteases ubiquitously found in animals and other organisms, which are classified into approximately a dozen families, like cathepsin A, cathepsin B, cathepsin C and others, based on their structure, catalytic mechanism and substrate specificity [60]. While many cathepsin proteases are thought to be mainly involved in intracellular protein turnover, remarkable cathepsin protease activities have been detected and characterized in the midgut of diverse hemiptaran, lepidopteran, coleopteran and other groups of insects, where they presumably function as digestive enzymes [61]–[64].

In this study, we found that several cathepsin L genes are highly and preferentially expressed in the midgut M4B region of symbiotic insects (Fig. 6A; Table S7), which was also confirmed by measuring cathepsin L activities in the midgut region (Fig. 6B). These results suggest that these cathepsin L genes play some biological roles in the M4B region of symbiotic insects. In the midgut of *R. pedestris*, the voluminous M4 region bears a number of crypts whose cavity is full of the symbiont cells, whereas the tubular M4B region is, although directly connected to the M4 region, devoid of crypts (Fig. 1) and exhibits much weaker symbiont signals than the M4 region [25]. Over-expression of cathepsin L protease genes in the M4B region may function to make substrates accessible to the symbiotic bacteria. Alternatively, the cathepsin L proteases in the M4B region may function to digest the symbiont cells overflowed from the adjacent M4 region, by which the host insect may control the symbiont population and/or utilize the symbiont-derived nutritional resources. Whether or not RNA interference of these cathepsin L genes results in accumulation of the symbiont cells in the M4B region will be a critical test for the hypothesis, which should be addressed in future studies.

In the aphid bacteriocytes, lysosomal activities are suggested to play important roles in controlling the obligate endosymbiont *Buchnera*, wherein lysosomes fuse to host-derived symbiosomes and degrade the symbiont cells therein [65]. Here it should be noted that not only lysosomal cathepsin proteases but also lysozymes and antimicrobial peptides are stored in endocellular granules [46], [60], which can be delivered to bacterial targets through membrane trafficking mechanisms: to endocellular bacteria via fusion to the symbiosome, and to extracellular bacteria via fusion to the cell membrane [53], [65]. In this context, we suggest that involvement of membrane trafficking for delivering effector molecules may underlie some molecular aspects commonly found among various endocellular and extracellular host-symbiont associations.

### Highly expressed ferritin genes in the midgut

Although neither related to the symbiotic insect nor to the M4 and M4B regions, it is notable that several ferritin subunit genes are highly expressed in the midgut of *R. pedestris* (Table S1). Ferritin is a ubiquitous globular protein of 450 kDa consisting of 24 subunits, which stores iron and releases it in a controlled fashion [66]. What biological roles the ferritin genes play in the midgut of *R. pedestris* is totally unknown, but, meaningfully, previous studies on diverse insects, crustaceans and nematodes reported that *Wolbachia* infections influence iron metabolism of their host organisms, affect host's fitness components in an iron-dependent manner, and up-/down-regulate ferritin gene expression [15], [16], [18], [67], [68].

### Genes related to innate immunity

The genome project of the aphid *A. pisum* revealed its peculiar innate immune system: while most insects possess three major immune gene cascades, the Toll pathway, the IMD pathway, and the JAK/STAT pathway [69], the aphid genome lacks IMD pathway genes, many antimicrobial peptides and c-type lysozyme [29], [70]. *R. pedestris* and *A. pisum* belong to the same insect order Hemiptera, but our EST analyses identified c-type lysozyme gene, defensin-like gene, and relish gene (Rped-1145, 1 clone) (Table S1) which is a key transcription factor in the IMD pathway [71], in *R. pedestris*. Hence, it is suggested that the lack of IMD pathway genes has evolved in the aphid lineage specifically.

### Effects of symbiosis on morphogenesis of the midgut symbiotic organ

Finally, we note that in the *Riptortus*-*Burkholderia* gut symbiosis, morphogenesis of the host symbiotic organ is remarkably affected by the symbiont infection: in the symbiotic insects, the midgut M4 and M4B regions become larger than those in aposymbiotic insects, while the midgut M3 region was smaller (Fig. 1). Enlargement of the M4 and M4B regions in symbiotic insects should reflect induction/suppression of many genes and functions involved in symbiosis, while enlargement of the M3 region in aposymbiotic insects may, although speculative, be due to resource allocation between the adjacent midgut regions. Because the morphological differences between the aposymbiotic midgut and the symbiotic midgut must have established before the fifth nymphal instar, gene expression for gut morphogenesis should be different at earlier developmental stages. The detailed observation on effect of symbiosis on midgut morphogenesis deserves future studies. Symbiont-induced morphogenesis of host symbiotic organ has been well documented in legume-*Rhizobium* nitrogen-fixing symbiosis [72], [73] and squid-*Vibrio* luminescent symbiosis [74], [75].

### Conclusion and perspective

In conclusion, using a conventional EST approach with relatively small amount of sequence data, we successfully identified some intriguing host genes that exhibit symbiosis-associated expression patterns in the *Riptortus*-*Burkholderia* gut symbiotic system. Recently, high-throughput next generation sequencing technologies have become readily available [76], which will enable much broader and deeper understanding of the host-symbiont interactions in a genomic/transcriptomic perspective. In *R. pedestris*, infection with the *Burkholderia* symbiont establishes at the second instar stage via oral ingestion [27]. Therefore, the EST analyses of fifth instar nymphs in this study must have unveiled consequences rather than processes of symbiotic influence on the host gene expression. In this context, transcriptomic comparisons between symbiotic and aposymbiotic insects at the post-infection, second-third instar stages are of interest. In *R. pedestris* and other heteropteran bugs, RNA interference generally works effectively [26], which provides a straightforward approach to functional understanding of the symbiosis-associated host insect genes. Future studies in these lines will shed light on the commonality and the diversity among various insect-microbe symbiotic systems ranging from bacteriome-specific obligate associations through systemic facultative associations to gut extracellular associations.

## Materials and Methods

### Insects and symbiotic bacteria

*R. pedestris* was collected from fields of the soybean *Glycine max* at Tsukuba, Ibaraki, Japan, and maintained in the laboratory. The locations are not privately-owned or protected in any way, and no specific permits were required. The field studies did not involve endangered or protected species. An isofemale line, TKS-1, was established and used for experiments. The insects were reared on soybean seeds and distilled water containing 0.05% ascorbic acid (DWA) at 25°C under a long-day regimen of 16 h light and 8 h dark. The *Burkholderia* symbiont strain RPE75 was used in this study, which is a spontaneous rifampin-resistant mutant derived from the strain RPE64 originally isolated from the midgut crypts of *R. pedestris* [27]. The symbiont was cultured with YG medium (5 g/l yeast extract, 4 g/l glucose, 1 g/l of NaCl) containing 10 mg/l of rifampicin (YG-RIF) at 150 rpm in broth or on 1.5% ager plates at 26°C.

### Oral administration of cultured symbiont

Hatchlings of *R. pedestris* were divided into two experimental groups: one was symbiotic (infected) group and the other was aposymbiotic (uninfected) group. In the aposymbiotic group, the nymphs were reared with symbiont-free DWA from hatching to fifth instar. In the symbiotic group, the nymphs were orally administrated with cultured *Burkholderia* symbiont as described [27]. The symbiont strain RPE75 was grown to an early log phase in YG-RIF medium on a gyratory shaker (150 rpm) at 26°C. Colony forming units (CFU) were estimated by plating the cultured media on YG-RIF agar plates. The symbiont cells were harvested by centrifugation, resuspended in DWA, and adjusted to 10^7^ CFU/ml. Each nymph was fed with the symbiont-containing water during the first two days of second instar stage. After the symbiont treatment, the water was replaced by symbiont-free DWA, and insects were reared until fifth instar.

### Construction of cDNA library and sequencing of EST clones

In order to construct cDNA libraries, a symbiotic insect and an aposymbiotic insect at the fifth instar were collected and dissected three days after molting. Three parts of the midgut (M3, M4B and M4) were dissected in phosphate buffered saline (PBS; 137 mM NaCl, 8.10 mM Na_2_HPO_4_, 2.68 mM KCl and 1.47 mM KH_2_PO_4_, pH 7.4), and total RNAs were immediately extracted from the tissues by using RNAiso plus (Takara), which were subjected to construction of cDNA libraries using SMART™ cDNA Library Construction Kit (Clontech) and Gigapack III Gold Packaging Extract (Agilent Technologies). The cDNAs ligated to λ phage vector were transformed with *Escherichia coli* BM25.8 (Clontech), in which the λ DNA was converted into a plasmid. The plasmids were amplified using Illustra Templiphi Amplification Kit (GE Healthcare) from a single colony of the *E. coli*, and sequenced using an ABI prism 3130 Genetic Analyzer (Applied Biosystems, Foster City, USA). All EST sequences have been deposited into the DDBJ database with accession numbers HX275191-HX282114. SignalP 4.0 [http://www.cbs.dtu.dk/services/SignalP/] was used for signal peptide prediction.

### Quantitative RT-PCR

Quantitative reverse transcription PCR (RT-PCR) was performed to evaluate the expression levels of candidate symbiosis-associated genes of *R. pedestris*. From each fifth instar nymph, four midgut parts (M1, M2, M3 and M4 [M4 + M4B]) were dissected in PBS, and total RNA was extracted by using RNAiso plus. The RNA samples were reverse transcribed with random primers (N6) and first-strand cDNA synthesis kit (GE Healthcare), and subjected to real-time quantitative PCR using a Stratagene Mx3000P (Stratagene, La Jolla, CA). Each of the PCR mixtures consisted of 2 µl of 10×TaqGold buffer (Applied Biosystems), 1.2 µl of 25 mM MgCl_2_, 2 µl of nucleotide mixture solution (2 mM each of dATP, dTTP, dGTP and dCTP), 0.2 µl of SYBR Green I (1/1,000-diluted solution) (Molecular Probes), 0.3 µl of primer mixture solution (10 µM each of forward and reverse primers), 0.1 µl of AmpliTaqGold DNA polymerase (Applied Biosystems), 8.9 µl of distilled water, 0.8 µl of dimethyl sulfoxide, and 4 µl of DNA sample solution. The PCR temperature profile was 94°C for 1 min, 35 cycles of 94°C for 1 min, 53°C for 1.5 min and 72°C for 1.5 min, followed by 72°C for 7 min. The primers are listed in Table S7. We used the standard curve method to calculate relative gene expression levels and used elongation factor 1 alpha (*EF1a*) gene of *R. pedestris* (accession number AB591382) as an internal control gene. For semi-quantitative RT-PCR, the cDNA samples were adjusted to the same concentration of *EF1a* cDNA copies using a Stratagene Mx3000P, and subjected to PCR amplification with the same primers.

### Assay of cathepsin L protease activity

Measurement of cathepsin L protease activity was performed as described [77]. Three parts of intact midgut (M3, M4B and M4) were dissected from symbiotic and aposymbiotic fifth instar nymphs, and individually homogenized in a lysis buffer (20 mM acetate buffer [pH 4.0], 50 mM NaCl, 5 mM EDTA, 5 mM 2-mercaptoethanol, 0.5% [vol/vol] Nonidet P-40). After centrifugation, 50 µl of the supernatant was combined with 445 µl of reaction buffer (0.1 M citrate buffer [pH 6.0], 75 mM NaCl, 5 mM EDTA, 2 mM cysteine), preincubated at 27°C for 5 min, and then mixed with 5 µl of 10 mM Z-Val-Val-Arg-MCA, a synthetic substrate for cathepsins L and S (Peptide Institute). After incubation at 27°C for 15 min, the reaction was stopped by adding 750 µl of 17% acetic acid. The protease activity was measured by a spectrofluorophotometer (RF-5300PC, Shimadzu) with excitation and emission wavelengths of 380 and 460 nm, respectively. As a negative control, samples were heat-inactivated at 95°C for 2 min prior to the enzymatic reaction.

### Molecular phylogenetic analysis

Amino acid sequences were aligned using Clustal_X [78]. Molecular phylogenetic analyses were conducted by three methods, neighbor-joining method using MEGA5 [79], maximum likelihood method using MEGA5 [79], and Bayesian with MrBayes v3.1.2 [80]. Bootstrap values for neighbor-joining and maximum likelihood phylogenies were obtained by 1000 resamplings. In total 7,500 trees were generated for each Bayesian analysis (ngen = 1,000,000, samplefreq = 100, burn in = 2,500).

## Supporting Information

Figure S1 **Molecular phylogenetic analysis of lysozyme genes.** A neighbor-joining phylogeny inferred from 1,380 aligned amino acid sites is shown, while maximum likelihood and Bayesian phylogenies exhibited substantially the same topologies. On each node, statistical support values are indicated in the order of [bootstrap value of neighbor-joining]/[bootstrap value of maximum likelihood]/[posterior probability of Bayesian]. Asterisks indicate support values lower than 50%. Red boxes indicate the *R. pedestris* genes.
(TIF)

Table S1 **List of nonredundant EST clusters obtained from midgut cDNA libraries of** ***R. pedestris*** **.**
(XLS)

Table S2 **List of EST clusters from** ***R. pedestris*** **representing either putative isoforms or premature transcripts.**
(XLS)

Table S3 **Assignment of Gene Ontology (GO) molecular function terms to the midgut EST data sets of** ***R. pedestris*** **.**
(XLS)

Table S4 **List of lysozyme genes.**
(XLS)

Table S5 **List of cysteine-rich secreted protein genes.**
(XLS)

Table S6 **List of cathepsin protease genes.**
(XLS)

Table S7 **Primer sets for quantitative RT-PCR.**
(XLS)
