# Supplementary figures and images for: Gene Expression in Gut Symbiotic Organ of Stinkbug Affected by Extracellular Bacterial Symbiont
Source: PLoS One. 2013 May 14;8(5):e64557. doi: 10.1371/journal.pone.0064557 (PMC3653873; doi:10.1371/journal.pone.0064557)

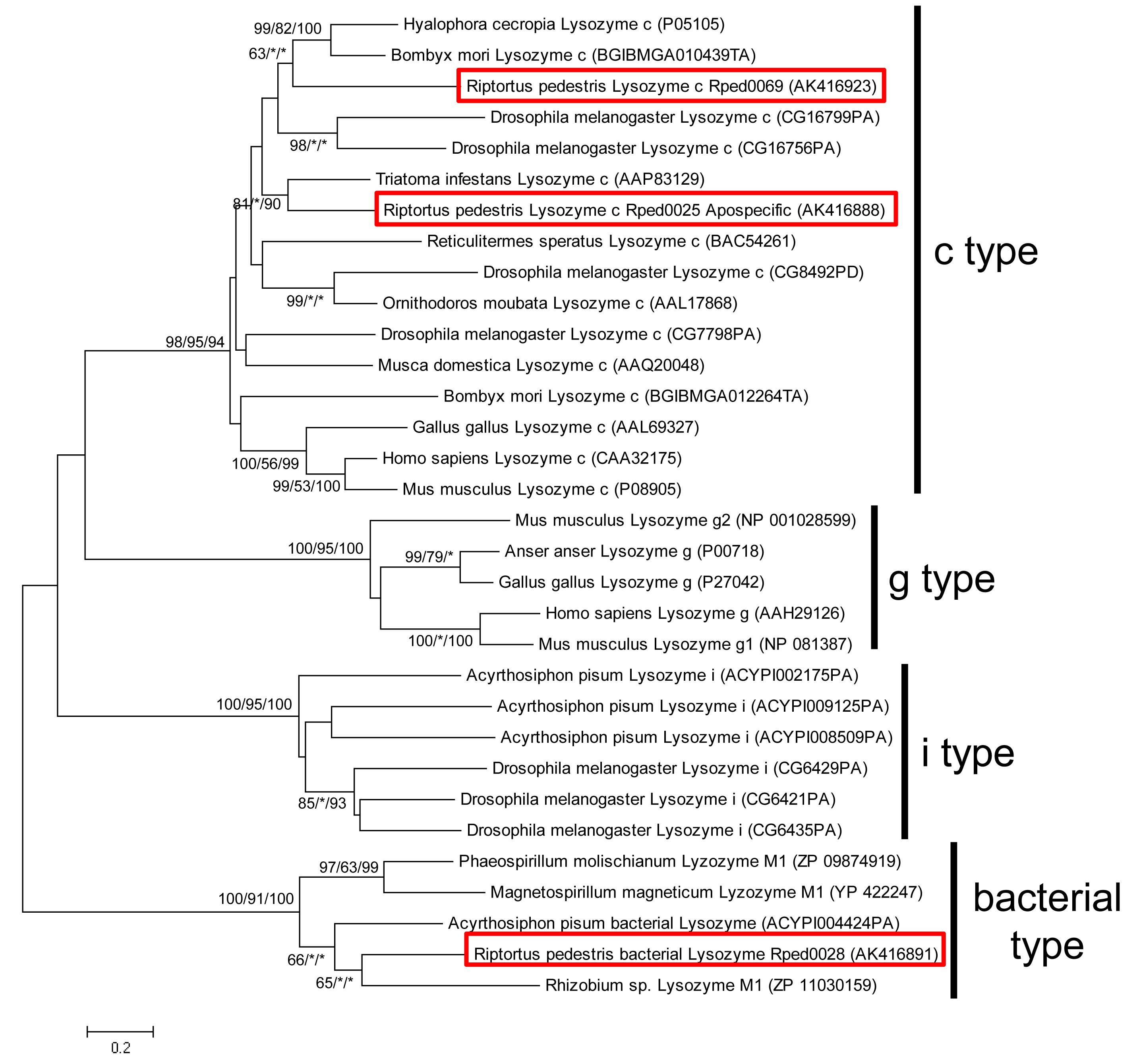

Supplement: Figure S1 — Molecular phylogenetic analysis of lysozyme genes. A neighbor-joining phylogeny inferred from 1,380 aligned amino acid sites is shown, while maximum likelihood and Bayesian phylogenies exhibited substantially the same topologies. On each node, statistical support values are indicated in the order of [bootstrap value of neighbor-joining]/[bootstrap value of maximum likelihood]/[posterior probability of Bayesian]. Asterisks indicate support values lower than 50%. Red boxes indicate the R. pedestris genes. (TIF) [file pone.0064557.s001.tif]
